# Behavioral health coaching for rural-living older adults with diabetes and depression: an open pilot of the HOPE Study

**DOI:** 10.1186/1471-2318-12-37

**Published:** 2012-07-24

**Authors:** Aanand D Naik, Craig D White, Suzanne M Robertson, Maria E A Armento, Briana Lawrence, Linda A Stelljes, Jeffrey A Cully

**Affiliations:** 1 VA HSR&D Houston Center of Excellence, Michael E. DeBakey VA Medical Center, 2002 Holcombe Blvd, Houston, TX, USA; 2 Department of Medicine, Baylor College of Medicine, Houston, TX, USA; 3 The University of Texas School of Public Health, Houston, TX, USA; 4 Department of Psychology, Texas A&M University, College Station, TX, USA; 5 Menninger Department of Psychiatry and Behavioral Science, Baylor College of Medicine, Houston, TX, USA; 6 VA South Central Mental Illness Research, Education and Clinical Center, Houston, TX, USA

**Keywords:** Diabetes, Depression, Goal-setting, Action planning, Telephone based health coaching, Geriatrics, Older patients

## Abstract

**Background:**

Patients with diabetes are at increased risk for depression, compounding the burden of disease. When comorbid with diabetes, depression leads to poorer health outcomes and often complicates diabetes self-management*.* Unfortunately, treatment options for these complex patients are limited and comprehensive services are rarely available for patients in rural settings.

**Methods:**

A small open trial was conducted to test the acceptability, feasibility and preliminary outcomes of a telephone-delivered coaching intervention for rural-dwelling older adults with uncontrolled diabetes and comorbid, clinically significant depressive symptoms. A total of eight older adults were enrolled in Healthy Outcomes through Patient Empowerment (HOPE), a 10-session (12-week), telephone-based coaching intervention. Primary study constructs included measures of diabetes control (Hemoglobin [Hb] A1c), depressive symptoms (Patient Health Questionnaire-9 [PHQ-9]), and diabetes-related distress (Problem Areas in Diabetes Scale [PAID]). Assessments were conducted at baseline, post-intervention, and 6-month follow-up. Acceptability and feasibility were evaluated using patient surveys, focused exit interviews, and session attendance data.

**Results:**

Clinically significant improvements were realized post-intervention and at 6-month follow-up for outcomes related to diabetes and depression. Effect sizes using Cohen's d were determined post-intervention and at 6-month follow-up, respectively, for HbA1c (*d=*0.36; *d=*0.28), PHQ-9 (*d=*1.48; *d=*1.67, and PAID (*d=*1.50; *d=*1.06) scores. Among study participants, HbA1c improved from baseline by a mean (M) of 1.13 (SD=1.70) post-intervention and M=0.84 (SD=1.62) at 6 months. Depression scores, measured by the PHQ-9, improved from baseline by M=5.14 (SD=2.27) post-intervention and M=7.03 (SD=4.43) at 6-month follow-up. PAID scores also improved by M=17.68 (SD=10.7) post-intervention and M=20.42 (SD=20.66) from baseline to 6-month follow-up. Case examples are provided for additional context and to more fully articulate salient intervention concepts.

**Conclusion:**

Although preliminary, data from this small open trial suggest that HOPE holds the potential to improve both physical (diabetes) and emotional (diabetes distress, depression) health outcomes and that changes can be maintained over a 6-month time period. As envisioned by the authors, HOPE may function as an extension of traditional primary care for rural-dwelling older adults with multiple comorbidities. A future randomized clinical trial will test HOPE’s broader effectiveness with rural-dwelling older adults.

**Trial registration:**

NCT01274715

## Background

Diabetes mellitus is a chronic disease affecting 8.3% of the US population, with this percentage sharply increasing for older adults (15-20%) and often leading to serious medical complications and high healthcare costs [1]. Patients with diabetes are at increased risk for depression, compounding the burden of disease [2]. When comorbid with diabetes, depression leads to poorer health outcomes and often complicates diabetes self-management [2-5]. Although multidisciplinary treatments for diabetes and depression are possible, access to such care is highly limited, especially for rural-dwelling older individuals [6,7]. It is far more common for patients with complex diabetes and depression to receive inadequate or uncoordinated treatment for these conditions [8-10].

Prior clinical trials for diabetes and depression suggest limited reach and modest effectiveness. Intensive programs involving depression medications and behavioral counseling (stepped care management, collaborative care) improve clinically significant depressive symptoms and quality of life in primary care patients with depression but not diabetes self-management or clinical outcomes [11]. Coordinated and stepped care interventions for comorbid cases appear to neglect diabetes care by relying on intervention models specific to depression management and directing intervention practices primarily toward depressive symptoms without translation to diabetes self-care [12,13]. A few studies currently under way utilize cognitive behavioral therapy, but these are again focused on either depression [14] or diabetes self-care [15] and not on behavior change techniques that can be applied to both conditions. Thus, behavioral strategies that activate patients to perform health behaviors for both diabetes and depression hold the potential to improve both the emotional and physical health difficulties of these medically complex patients*.*

The current study used a small open trial format to examine the acceptability, feasibility and preliminary outcomes of a telephone-delivered behavioral coaching intervention for rural-dwelling older adults with uncontrolled diabetes and comorbid, clinically significant depressive symptoms. The intervention used a flexible patient-centered approach to treatment, targeting physical and emotional health issues simultaneously.

## Methods

### Procedure

All procedures for the HOPE trial were approved by the Baylor College of Medicine Institutional Review Board (IRB) and the Michael E. DeBakey Veterans Affairs (VA) Medical Center Office of Research and Development, Houston, TX.

#### Participants

HOPE recruitment sought to engage rural-living older adults with uncontrolled diabetes and comorbid, clinically elevated depressive symptoms. Data from a VA patient registry was extracted to identify rural-dwelling individuals with uncontrolled type 2 diabetes mellitus, as indicated by average glycated hemoglobin (HbA1c) of 7.5 or greater and no single HbA1c marker below 7.0 over the past year. Individuals who met these inclusionary criteria were mailed an opt-out letter describing the HOPE Program. One week after the mail-out, a member of the project team called potential participants to ascertain their interest in participating. Interested individuals completed a short screening assessment, including a six-item cognitive screener, a three-item mental health screener, and the Patient Health Questionnaire-2 (PHQ-2), a validated, short version of the PHQ-9, used to screen for depressive symptoms [16]. A binder with detailed study information, including informed consent and print-based aids for telephone assessments, was sent to each person who met initial screening criteria. A project team member then called qualifying patients to review consent materials and obtain audio-taped consent prior to conducting a baseline assessment interview, which included a demographic questionnaire and the PHQ-9. Patients satisfying baseline depression screening requirements (i.e., PHQ-9 score of 10 or greater) were enrolled in the HOPE program, administered the remaining baseline assessment measures, scheduled for lab work (i.e., HbA1_C_ test) at their local primary care site, and mailed a HOPE program intervention workbook.

#### Intervention

The HOPE intervention consisted of ten 30–45 minute sessions delivered by telephone over a 12-week period. The overall objective of HOPE was to assist participants to improve their ability to self-manage their physical and emotional health. HOPE coaches (described below) sought to assist participants to prioritize their self-care needs, and to identify and resolve barriers to effective self-care. Participants learned and practiced skills that would help them reach the goals and action plans they set for themselves to improve their self-care [17]. While prior clinical interventions have targeted either physical or emotional issues, HOPE extends these efforts by addressing both dimensions simultaneously [18].

HOPE uses a structured patient workbook to guide participants and coaches. The workbook, modeled using prior work from this group [19,20] used a module-based approach where participants and coaches selected treatment goals and self-management skills that met the individual needs of the patient. The workbook was structured to facilitate behavioral change through collaborative goal-setting and action planning across physical and emotional health domains. It was ideographic by nature, allowing coaches to use structured information presented in physical and emotional health modules to stimulate conversation around issues of personal salience to patients, to educate patients on these issues and to build skills necessary to practice goals and action plans. Goal-setting and action planning components (e.g., worksheets to stimulate collaborative construction of high-quality goals and action plans) were incorporated throughout physical and emotional heath modules. These components were previously employed in a randomized clinical trial demonstrating that collaborative goal-setting in group diabetes clinics is associated with improved diabetes control [20]. In the HOPE intervention, collaborative goal setting and action planning reflected an iterative process whereby coaches assisted patients to set personally meaningful goals and action plans to meet their goals. Coaches continuously assessed goal attainment with patients, focusing on barriers and collaborative problem-solving to negate these barriers.

The coaching model for the HOPE Program was based on the 5 *A*s Model for coping with chronic illness [21,22]. Guided by the 5 *A*s (i.e., Assess, Advise, Agree, Assist, Arrange), intervention coaches assisted participants in selecting and refining physical and emotional health goals and in developing action plans to meet their goals.

### HOPE sessions and modules

Table 1 describes the content of session modules and the overall intervention structure by session. HOPE was structured around core concepts but also included a modular component that allowed coaches and patients to focus on physical and mental health difficulties of personal salience to the participant. The goal of this modular-based approach was to advance patient involvement and empowerment through active participation in the treatment process.

**Table 1:** HOPE intervention session structure and module content

**Session#**	**Week**	**Session Content**	**Session Goals**
**Core Sessions**			
**Session 1**	**1**	**Getting to Know HOPE and Your Coach**	
		·General introduction to the HOPE Program	·To build rapport between participant/coach
		·Outline of program structure and module content	·To encourage open discussion
		·Definition of roles/expectations of participant and coach are defined	·To customize a program to participants’ needs
**Session 2**	**2**	**Setting Goals and Making Action Plans**	
		· Introduction to the concept of goal setting and action planning	· For participant to have a foundational understanding of goal setting/action planning
		· 3 rules to follow in developing a high-quality goal	· To make first attempt at setting a high -quality goal
		· Action Plan Checklist	
**Elective Sessions**			
		**Module A: Managing Your Medications**	
		· Knowing Your Medications	· For participant to better understand medication outcomes (benefits vs. side-effects)
		· Choosing the Right Medication for You	· To build skill: improve communication with PCP/healthcare team
		· Keeping a Schedule for Taking Your Medications	· To set/adjust a medication-adherence goal
		**Module B: Using Thoughts to Improve Wellness**	
		· Recognizing How Thoughts Affect Mood and Behaviors	· For participant to understand the impact thoughts have on feelings and actions
		· Using Coping Statements to Improve Wellness	· To build skill: coping, alternative thinking
		· Increasing Positive Thinking/Decreasing Negative Thinking	· To set/adjust a goal for practicing thought- monitoring strategies
		**Module C: Eating a Healthy Diet**	
		· Controlling Carbohydrate Intake	· To improve participant’s knowledge of nutrition and dietary recommendations
		· Increasing Fruit And Vegetable Intake	· To build skill: balanced food-group servings, portion control, healthy food substitutions
		· Reducing Unhealthy Fat Intake	
		· Limiting Portion Sizes	· To set/adjust a healthy diet goal
***Sessions 3-8***	***3-8***	**Module D: Increasing Pleasant Activities**	
		· Recognizing How Behaviors Affect Thoughts and Moods	· For participant to understand the impact actions can have on thoughts and feelings
		· Identifying Meaningful Activities	· To build skill: resuming or beginning new activities of interest
		· Engaging In Pleasant Activities and Being Active	· To set/adjust a behavioral activation goal
		**Module E: Getting Fit**	
		· Improving Endurance Through Cardiovascular Exercise	· To educate participant about recommended exercise guidelines
		· Improving Flexibility Through Stretching	
		· Improving Strength and Muscle Tone Through Strength Training	· To set/adjust a fitness goal, considering participant’s physical limitations
		***Module F: Learning How to Relax***	
		· Understanding What Stress, Worry, and Anxiety Are	· To identify stressors that impact health
		· How to Use Deep Breathing to Reduce Stress and Tension	· To build skill: practicing deep breathing and/or imagery exercises
		· How to Use Imagery to Reduce Stress and Tension	· To set/adjust a goal for incorporating relaxation techniques into participant’s daily routine
***Follow-Up Sessions***			
***Session 9***	***10***	***Adjusting Your Action Plans and Overcoming Obstacles***	
		· Review all previous goals and assess goal progress	· For participant to reflect on accomplishments realized through the HOPE Program
		· Discussion of remaining barriers/obstacles to goal success and identification of strategies for overcoming them	· To build skill: overcoming barriers/obstacles
			· To make final adjustments to Action Plans
***Session 10***	***12***	***Managing Your Resources and Final Discussions***	
		· Final discussions:	· For participant to reflect on diabetes-care skills acquired in HOPE
		· Diabetes ABCs	· To prepare participant to continue goal development on his/her own, following HOPE
		· Resources/support system	· For participant to be satisfied with program outcomes and committed to achieving long-term health goals
		· Monitoring Action Plans/tracking goal progress	
		· Closing remarks	

#### Core sessions (sessions 1 and 2)

The first two HOPE sessions educated participants about the importance of holistic diabetes self-care, emphasizing both physical health monitoring and emotional well-being as complementary dimensions of maintaining overall health. Session 1 introduced participants to the HOPE intervention and focused on rapport building by assessing feelings about diabetes and physical and emotional health challenges. In Session 2, participants learned the principles of effective goal setting and action planning and begun to apply these theoretical principles to their own situations.

#### Elective sessions (sessions 3 to 8)

HOPE provided participants with the flexibility and autonomy to select from six elective physical and emotional health modules. Elective modules included: Managing Your Medications, Using Thoughts to Improve Wellness, Eating a Healthy Diet, Increasing Pleasant Activities, Getting Fit, and Learning How to Relax (*see* Table 1). Each module incorporated skill-building techniques, didactic learning, and interactive exercises designed to facilitate goal setting and action planning. Participants chose the order in which modules were discussed, based on their preferences and self-care needs. Participants could spend two or more sessions focused on a particular module, either to build on a successful attempt at implementing an action plan, or to revise an action plan he/she was unable to practice because of unforeseen obstacles.

#### Follow-up sessions (sessions 9 and 10)

In Session 9, coach/participant dyads reviewed all goals developed in prior sessions and made final action-plan adjustments, as necessary. Session 10 focused on maintaining changes post-treatment.

### HOPE coaches (providers)

Given the limited availability of mental health providers in the current medical care environment, HOPE sought to demonstrate the appropriateness of using non-expert mental health coaches. Coaches were recruited from a variety of disciplines and training levels including a clinical psychology graduate intern, a developmental psychology postdoctoral fellow, a doctoral student in public health, and a post-bachelors psychology student. A large body of literature supports the role of paraprofessionals, community health workers, and peers in the delivery of self-management coaching [23-25]. While none of the coaches in the current study had focused training in diabetes care, and only one coach (psychology intern) had experience treating depression, all coaches received structured training and follow-up assessment by qualified clinicians.

Coaches also interfaced with primary care providers and the broader clinical care setting using the VA’s electronic medical records (EMR) system. Coaches accessed clinical patient information from the EMR to review past medical history and identify ongoing treatment plans from the primary care team in order to set coordinated clinical care goals and action plans that enabled the established treatment plan. Coaches then used standardized note templates to inform PCPs about their patients’ goals and action plans. Additionally, coaches encouraged patients to communicate any further concerns to their PCPs by assisting them to set goals and action plans around doctor-patient communication. In essence, HOPE coaches functioned as clinician-extenders rather than an alternative or auxiliary provider.

### Intervention fidelity

Coaches were trained and provided with ongoing support for the intervention, structured around the HOPE workbook and larger intervention materials. All coaches received initial and ongoing training (and monitoring of cases) in the HOPE procedures via weekly group and individual supervision lead by the intervention’s dual principal investigators, a medical geriatrician (Naik) proficient in primary care of diabetes and a licensed clinical psychologist (Cully) who specializes in treatment of mental disorders and comorbid chronic disease. During supervision meetings, coaches would review patients’ progress and address challenges related to intervention concepts and use. Coaches also learned from their peers through discussion of issues that commonly emerged across patients (e.g., reluctance to address medication management), including the generation of potential solutions that could be used in subsequent sessions.

In accordance with standardized clinical and research protocols, coaches were specifically trained to respond to crisis situations should a patient present with suicidal ideation/intent or notable decompensation in their physical or emotional health. A suicide protocol approved by the IRB included steps to be followed by a coach should a patient report suicidal ideation or other clinical crisis information. This protocol included a cascade list of licensed mental health and medical providers to be contacted immediately by the coach in the event of a high-risk situation. Licensed providers with hospital privileges would follow up by calling the patient, assessing their emotional and physical health needs, and responding with appropriate care recommendations.

### Measures

All study measures were collected by a trained interviewer who remained blind to the intervention practices of the open trial.

#### Glycated Hemoglobin (HbA1c)

HbA1c is a form of hemoglobin measured in lab testing to evaluate an individual’s average percentage of blood glucose concentration over a 3-month period. HbA1C is the gold standard for evaluation of diabetes control, as defined by The American Diabetes Association’s recommendation of HbA1c of 7% or less [26].

#### Depression

The PHQ-9 is a well-validated assessment of depressive-symptom severity and is widely used within the VA [26]. Test-retest reliability of this measure demonstrates its diagnostic utility and capacity to measure significant change (sensitivity to change) in clinical settings [27,28]. Nine items determine the presence and degree of all *Diagnostic and Statistical Manual, Fourth Edition* criteria on a Likert-type scale, ranging from 0 (“not at all”) to 3 (“nearly every day”). Total scores≥ 10 represent clinically significant depressive symptoms and inter-test score differences≥ 5 suggest clinically significant change [27,28].

#### Diabetes distress

The Problem Areas in Diabetes Scale (PAID) was used to assess diabetes-specific emotional distress [29,30]. This 20-item scale measures the extent to which living with and managing diabetes contributes to feelings of emotional burden (e.g., guilt, frustration, anger, depression). Response options range from 0 (“not a problem”) to 4 (“serious problem”). Scores are transformed on a scale from 0 to 100, with higher scores reflecting greater distress. Prior research suggests that a score of 50 or greater indicates clinically significant diabetes-related distress [31].

## Results

Eight patients with a mean age of 62 years (range=58–67) were enrolled in the program. Following enrollment, an intervention coach was assigned. Intervention coaches called their assigned participants within 5 days of mailing the participant workbook to schedule their first HOPE session.

The following results section will present clinical outcome data from the HOPE trial followed by three case study reports that provide additional information about salient intervention concepts. All participant names have been changed.

### Quantitative data

Eight older individuals enrolled in HOPE, seven of whom completed the program (one did not proceed beyond session 1 because of an unrelated hospital admission). Enrolled participants were, on average, 62 years of age (range 58–67), had been living with diabetes for over 17 years, and lived over 30 miles from their primary care provider (PCP). Over 70% of the participants had received mental health treatment in the past, and over 85% were receiving insulin therapy. Table 2 contains a full listing of participant demographic characteristics. Clinical outcome data from this trial, including HbA1c, PHQ-9, and diabetes distress scores pre- and post-treatment, can be found in Table 3. Effect sizes using Cohen's d (with pooled standard deviations) were found for HbA1c at post-treatment (*d=*0.36) and 6-month follow-up (*d=*0.28); PHQ-9 at post-treatment (*d=*1.48) and 6-month follow-up (*d=*1.69); and PAID at post-treatment (*d=*1.50) and 6-month follow-up (*d=*1.06).

**Table 2:** Baseline characteristics of the study population

***Characteristics***	***Value***
***Age in years,****M (SD)*	62.1 (2.85)
***White race,****n (%)*	4 (57)
***Male gender,****n (%)*	6 (86)
***Body mass index (BMI),****M (SD)*	34.1 (6.47)
***Years living with diabetes,****M (SD)*	17.4 (7.39)
***Distance to PCP (in miles),****M (SD), R*	33.6 (17.6), 15-70
***Prior mental health treatment,****n (%)*	5 (71)
***Receiving depression treatment,****n (%)*	4 (57)
***Receiving insulin therapy,****n (%)*	6 (86)

*M* Mean, *SD* Standard Deviation, *R* Range, *n* number of participants, *PCP* Primary Care Provider.

**Table 3:** Clinical outcome data for the HOPE Pilot study cohort

***Data-Collection Timeframe***	***Outcome Measures***		
	***HbA1c***	***PHQ-9***	***PAID***
***Baseline, M (SD)***	9.73 (2.62)	14.6 (2.99)	46.8 (6.29)
***3-month, M (SD)***	8.60 (3.56)	9.43 (3.91)	28.9 (15.6)
***Change from baseline to 3-month, M (SD)***	1.13 (1.70)	5.14 (2.27)	17.7 (10.7)
***Effect Size, Cohen’s d***	0.36	1.48	1.50
***6-month, M (SD)***	8.89 (3.35)	7.57 (5.03)	26.9 (25.9)
***Change from baseline to 6-month, M (SD)***	0.84 (1.62)	7.03 (4.43)	20.4 (20.7)
***Effect Size, Cohen’s d***	0.28	1.69	1.06

*M* mean, *SD* standard deviation, *R* range;

Effect sizes measured using Cohen’s *d* (0.8=large effect, 0.5=medium effect, 0.2=small effect).

*Baseline=*readings at baseline assessment;

*3-month=*readings at 3-month follow-up assessment;

*6-month=*readings at 6-month follow-up assessment.

*HbA1c* hemoglobin A1c; Lower HbA1c is consistent with better diabetes control.

*PHQ-9* Patient Health Questionnaire-9; Higher total PHQ-9 scores indicate elevated depressive symptoms.

*PAID* Problem Areas In Diabetes scale; Higher total PAID scores represent greater diabetes related distress.

Upon completion of the coaching sessions, exit interviews were conducted with each participant, rating items on a 5-point Likert scale (0=not at all, 5=very helpful). On average, participants’ responses to the following items suggested strong acceptability of the HOPE program: “Overall, how would you say the HOPE program was in improving your quality of life?” (*M=*4.5, *SD=*0.756); “Overall, how would you say the HOPE program was in helping you to learn to manage your diabetes?” (*M=*4.625, *SD=*0.744); “Overall, how would you say the HOPE program was in helping you to manage stress, anxiety, and depression?” (*M=*3.375, *SD=*1.506); and “Overall, how confident are you that you will continue to use the skills in the future?” (*M=*4.5, *SD=*0.535, where 5=very confident).

### Qualitative data (case study reports)

The narratives below introduce HOPE patients and illustrate coaches’ use of the 5 *A*s model of coping within a chronic illness framework including focused intervention efforts related to goal setting and action planning.

#### Case 1: Brian

**Case 1: Brian, age 63.** Brian, a retired Veteran, reported high diabetes interference in his daily life at baseline interview. His HbA1c, which ranged from 8.9 to 11.3 over the last year (*M=*10.9, *SD=*1.19), was 11.2 at baseline. He was also morbidly obese, suffered from sleep apnea, and had been diagnosed with Major Depressive Disorder, exemplified by his PHQ-9 baseline score of 13. He acknowledged that his diet was poor and that he had limited diabetes knowledge. Brian was admittedly doubtful at the start of the intervention of HOPE’s potential efficacy in producing positive health outcomes.

After assessing Brian’s diabetes knowledge and concerns about the HOPE Program, his coach advised him on goal-setting. Acknowledging his unstable HbA1c markers, Brian agreed to set multiple diet goals, including controlling meal portion sizes, increasing fruit and vegetable intake, reducing carbohydrate intake, and keeping a food log to monitor this goal for the duration of the program. Brian was initially skeptical about the process; however, as he began successfully reaching goal-setting milestones, he became increasingly engaged in the program. Brian experienced significant goal attainment, consistently increasing the scope of his goals as he progressed, and later adding fitness and medication-adherence goals. His coach assisted him in improving communication with his wife and increasing his diabetes care self-efficacy, arranging a self-monitoring plan to help maintain future success and communication action plans with his PCP to help manage mild hypoglycemia symptoms with improved control. Brian realized a marked drop in HbA1c from 11.2 at baseline to 7.2 at 6-month follow- up, and a reduction in diabetes distress (PAID score improved 3.75 at 6-month follow-up). Though his depression score at baseline (13) showed clinically significant improvement at 3-month follow-up, his depressive symptoms at 6-month follow-up returned to baseline functioning, suggesting the need for additional depression intervention.

#### Case 2: Laura

**Case 2: Laura, age 59** Laura was a disabled Veteran, divorced, and living alone. She entered the HOPE program with severely high HbA1c at baseline (14.8), shown to be consistent over the past year (*M=*13.5, *SD=*2.15). She disclosed that daily diabetes monitoring limited her physical activity, causing stress and anxiety. In addition, she suffered from multiple comorbidities, including Major Depressive Disorder (demonstrated by her high baseline PHQ-9 score of 19), post- traumatic stress disorder, and asthma. Prescribed an extensive list of medications, she admitted to having trouble adhering to her complicated regimen because of side-effects, financial limitations, and scheduling conflicts.

During her initial session, Laura expressed optimism for the potential outcomes of HOPE, despite some initial resistance to participate because of failures experienced in a prior diabetes education program. On the basis of Laura’s extremely high diabetes numbers, her coach advised her to take immediate steps to alter lifestyle choices; she agreed to begin walking for 30 minutes daily, planning to increase scope and potency of this goal as she progressed. She also committed to stop eating fast food and began packing healthy snacks to carry throughout her day. However, after completing her fourth session, she experienced a stressful life event that caused her to disengage from HOPE, and she began missing and/or rescheduling coaching sessions. Given the significant barriers to goal attainment, Laura and her coach decided not to attempt any new goals. Her coach attempted to assist her to overcome barriers to her initial goals. They used the remaining interactions to arrange a prolonged monitoring plan and, although Laura verbally maintained commitment to finish the program, she eventually completed only five sessions. Though she reported improvements in diabetes distress at 3-month follow-up (PAID score improved by 30) and depression at 3-month follow-up (PHQ-9 score had significant improvement of 6 points), her HbA1c did not improve (increase from 14.8 at baseline to 16.1 at 6-month follow-up). By the conclusion of the pilot, her PCP had ordered a more aggressive medical intervention; yet she maintained her positive improvements related to depressive symptoms and diabetes distress.

#### Case 3: Daniel

**Case 3: Daniel, age 62** Daniel was a disabled Veteran, divorced, and living alone at the time of treatment initiation. At baseline, he reported feeling stuck in daily routines (e.g. poor diet, lack of exercise) that he considered unhealthy. He also reported problems falling and staying asleep, which interfered with the daytime activities necessary to effectively manage his diabetes. His moderately high baseline markers for HbA1c (8.8) and PHQ-9 (14) were likely a result of these factors. He felt a great deal of stress and worry over potential complications of his condition, especially because several of his family members had experienced diabetes-related complications. Additionally, he found social situations that involved eating to be difficult, given his dietary restrictions, and admitted to occasional interpersonal difficulties caused by low frustration tolerance and poor stress management.

In assessing Daniel, his coach recognized lifestyle problems common to uncontrolled diabetes and advised that simple lifestyle modifications would make a big difference in overall well-being. The dyad agreed to begin with a fitness goal of walking ¼ mile 5–6 days per week, which Daniel successfully increased to 1 mile as he progressed through the program. In addition to addressing physical goal barriers, such as pain from a leg injury that caused sleep problems, Daniel’s coach assisted him by helping him identify emotional concerns that could impede goal attainment. Daniel became open to practicing relaxation techniques during coaching sessions and established a goal to incorporate them in his daily life. In arranging for prolonged goal maintenance, his coach emphasized the need to identify barriers to his goals and encouraged him to share his diabetes experiences with others to help improve his social connections. At the conclusion of the treatment, Daniel realized notable 6-month changes, including moderate yet clinically significant improvement in HbA1c (drop of 0.9 from baseline) and marked reductions in depression (PHQ-9 score improved 14 points) and diabetes distress (PAID score improved 37.5 points).

## Discussion

The HOPE program is a structured, theory-driven psychosocial intervention that uses telephone-based behavioral health coaching to promote lifestyle changes for improved diabetes control and reduced depressive symptoms in older rural-dwelling patients. Initial data from the HOPE program suggest that the multifaceted intervention, as designed, may generate positive outcomes for both physical and emotional aspects of diabetes self-care. Patient-level data and qualitative interviews post-treatment suggest that patients responded positively to the collaborative goal setting and action-planning process. Prior diabetes and depression interventions have typically shown positive outcomes for either diabetes or depression markers, but not both [32-39]. Under these more narrowed aims, structured behavioral intervention strategies have been demonstrated to be more effective at improving glycemic control than traditional diabetes education programs in patients with diabetes for at least two years [40]. In contrast, the HOPE program demonstrated an ability to cultivate positive outcomes across all clinically relevant measures, including diabetes-related distress, in a group of medically complex patients with an average diabetes duration of 17 years. Changes in clinical outcomes may be related to reductions in diabetes-related distress, measured by the validated PAID instrument [41]. These findings, derived through the application of a simple telephone-based protocol delivered by non-expert coaches, extend the results seen in more resource-intensive interventions targeting diabetes and depression [42].

Common among the HOPE pilot cohort was the initial selection of more traditional diabetes lifestyle goals (e.g. diet and exercise), rather than emotional health goals. On the whole, participants were initially reluctant to discuss topics related to emotional well-being, possibly because they did not want to appear vulnerable, or due to lack of awareness and understanding of a potential connection to self-management and physical health. Those participants who did engage around affective goals were usually encouraged to do so by their coaches when they encountered goal barriers that could not be overcome through physical health changes alone. It may have been this private individualized attention from coaches and the built-in process of building rapport that eventually encouraged goal-setting around emotional health issues. Prior research has shown individual education to be superior over group education and usual care in reducing HbA1c, further supporting this notion [43]. Importantly, outcome data from the trial suggest that, although emotional health concerns were not always addressed overtly, especially at the onset of treatment, depressive symptoms may be reduced, as evidenced by the 6-month effect size for PHQ-9 scores (*d=*1.70).

Participants were also generally hesitant to discuss their diabetes medication regimens including associated side-effects, unless hypoglycemia-related issues arose during the program. If this happened, coaches helped participants to identify and monitor hypoglycemia-related events and construct action plans with PCPs to address this potentially dangerous side-effect. Thus, the HOPE program may be an effective method of addressing hypoglycemia-related events that arise during diabetes interventions.

The pilot cohort completion rate indicated high patient acceptability and satisfaction with the HOPE intervention. HOPE holds the potential to be implemented in clinical practice and the use of non-experts as coaches may reduce potential "translational" difficulties including limited mental health provider availability and other time and resource limitations common to the use of behavioral change interventions. HOPE may be especially suitable for older adults with multiple chronic conditions. The telephone-based nature of the program increases access to care for vulnerable rural-dwelling individuals who may be unable or unwilling to travel to and from a central treatment site due to functional limitations or a lack of social support [44]. Importantly, HOPE was structured to support and sustain aging in place initiatives that promote independent living among older adults. The HOPE protocol also fosters self-efficacy with chronic self-management by encouraging, supporting, and providing practice for participants to engage in proactive coping and skill development efforts that initiate and sustain behavioral change. Coping skills prevent and alleviate stress allowing older adults to preserve resources that are susceptible to aging-related losses and declines [45]. Previous research also highlights the importance of perceived control as a key component of successful aging [46,47]. Our study corroborates previous research [48] suggesting that middle-aged and older adults can learn, integrate, and practice proactive coping skills. Using these skills, it appears that the rural-dwelling adults in our study were able to take charge of their own health and better position themselves for future health challenges.

### Limitations and future research

A number of limitations in the present study should be acknowledged. While participants in the HOPE trial realized notable positive clinical changes, the small sample size and lack of control group limit the generalizability and interpretation of study clinical outcomes. Although effect sizes were maintained at 6-months, time constraints to complete the pilot did not allow for longer-term follow-up sessions to promote maintenance of goal attainment. A larger trial could allow for a more comprehensive examination of the HOPE intervention using a larger sample size, intervention control group, and lengthier follow-up to evaluate long-term self-management behaviors.

It is important to note that the age cohort (range=58–67) of our pilot participants may have led to greater intervention acceptability and better clinical outcomes than would be realized with an older age group. However, due to the complexity and severity of conditions experienced by this cohort at baseline, our results show promise for the potential of clinical improvements in others treated within the HOPE program.

Lastly, we did not seek direct PCP engagement in the treatment process, although coaches did communicate goal progress through passive notes in patients’ electronic medical records and were aware of PCPs’ treatment plans as documented in medical records. Increased interface with participants’ PCPs above the standard chart notes could increase provider engagement and may have helped guide tailored intervention plans for each patient potentially further personalizing treatment. We acknowledge there are potential difficulties inherent in engaging clinicians, particularly clinician availability and willingness to participate. Future work should focus on developing a system that would further facilitate sharing of information between PCPs and HOPE coaches.

Experiences from the HOPE open pilot suggest that patients with severe depressive symptoms and/or long-standing mental health issues, such as Major Depressive Disorder or post-traumatic stress disorder, may be poor candidates for HOPE, as they require mental health treatment beyond the scope of this intervention. Future studies should examine the association between severity of depression and other mental health comorbidities in response to treatment and potential need for a stepped-care approach.

## Conclusion

Overall, HOPE demonstrated clinically meaningful improvements in diabetes control, depressive symptoms, and diabetes distress over a 6-month period, using a tele-coaching intervention. HOPE appears to be a feasible and potentially cost-effective, telephone-delivered coaching intervention targeting high-risk older patients with comorbid diabetes and depression. Because older adults experience increased obstacles (e.g. financial limitations; lack of transportation/inability to self-transport; comorbid/unrelated health conditions restricting mobility) to participation in clinic-based diabetes protocols, HOPE may provide a viable option to reach geographically isolated or disparate populations with limited access to comprehensive diabetes and mental health care. While HOPE cannot replace the need for basic primary care, it does provide a potentially critical adjunct for aging patients with treated but uncontrolled diabetes and depression. A larger study with lengthier follow-up and greater PCP engagement is needed to support and extend the findings of this pilot.

## Competing interests

The authors declare that they have no competing interests relating to the HOPE project or this manuscript.

## Authors’ contributions

The authors’ responsibilities were as follows- AN and JC: conception and design of intervention, drafting and revisions to the article. CW: behavioral coaching, drafting and revisions to the article. MA, SR, and BL: behavioral coaching and revisions to the article. LS: study coordination and revisions to the article. All authors read and approved the final manuscript.

## Pre-publication history

The pre-publication history for this paper can be accessed here:

http://www.biomedcentral.com/1471-2318/12/37/prepub
